# The Hospital. Nursing Section

**Published:** 1902-08-30

**Authors:** 


// The Hospital.

**Nursing Section.**

Contributions for this Section of "The Hospital" should be addressed to the Editor, "The Hospital" Nursing Section, 28 & 29 Southampton Street, Strand, London, W.C.

No. 831.—Vol. XXXII. *SATURDAY, AUGUST* 30, 1902.

### Notes on News from the Nursing World.

### THE NEW MATRON OF UNIVERSITY COLLEGE HOSPITAL.

The appoinment of Miss Dora Finch to the post of matron of University College Hospital, in succession to Miss Hamilton, matron designate of St. Thomas's Hospital, is only to be regretted because it deprives the New Hospital for Women of an admirable head of the nursing staff. Miss Finch, who merely moves from Euston Road to Gower Street, was trained at St. Bartholomew's Hospital and was subsequently sister in the same institution. She has been matron at the New Hospital for Women since July, 1899, during which period she has shown administrative abilities of a high order, and in a more onerous position she will, doubtless, be found fully equal to her responsibilities.

### NURSES FROM SOUTH AFRICA.

The nurses who have lately returned from South Africa, owing to the fact that their services are no longer required, include: On the *Salisbury*, Sisters J. A. Davies and A. J. Wylie; on the *Saxon*, Sisters R. M. Browse and L. Hand; on the *Tintagel Castle*, Sisters A. J. Davidson, F. Grayston, A. L. Von-Dix, A. E. Andre; on the *Simla*, Sisters S. A. Boucham, R. A. Chew, M. T. Gilmour; on the *Orissa*, Sisters A. D. Cameron, A. R. Mackintosh, A. A. S. Hill; and on the *Dilwara*, Sisters J. C. Spence, E. M. Pethick, E. Newton, and L. Parsons. Superintendent Sister J. A. Gray, and Sisters G. E. Saunders, A. A. C. Higgs, and Wilson have also arrived, but return. The following are described as invalids: Sisters M. Rickards, G. E. Larmer, W. M. Pughe, and H. A. Hayhurst.

### ARMY NURSES IN THE UNITED STATES.

The order issued by the United States War Department in reference to the Army Nurse Corps, established last year, fixes the period for the appointment of nurses at three years. No nurse can be admitted to the corps who is not a graduate of a hospital training school. After her appointment, a nurse has to serve for three months in the United States, during which time she will receive special instruction in army nursing. When she is on active service her pay and allowance will be $40 a month in the States and $50 abroad. She will be furnished with quarters and will receive one ration in kind per day. When she is stationed where rations cannot be furnished, she will receive commutation of rations at 75c. per day. The total duration of leave of absence with pay will be 30 days, and an additional month without pay will be granted when the service will permit. The compulsory age of retirement is 45.

### THE WOMEN S MEMORIAL TO QUEEN VICTORIA.

The contribution of the Pontypridd and East Glamorgan districts towards the National Women's Memorial to Queen Victoria is £206 16s. 1d. An interesting feature is that this sum is made up of small amounts from various districts, ranging from Rudry 17s. to Pontypridd £57 9s. 9d. No fewer than twenty-four districts are represented. In this part of the Principality, as elsewhere, the movement has been supported with remarkable enthusiasm by the working classes.

### DISTRICT NURSES AND FUSSINESS.

At the half-yearly meeting of the Darwen Nurses' Association, Mr. Slater said that he had heard many people complain of the fussiness of the nurses. When they came into a house, he said, they wanted first one thing and then another doing for them. "Working people," he continued, "had not time to fuss about in this way. He could quite understand that the nurses wanted to keep the patients clean, but he thought that sometimes they did it at the peril of the sick person. He thought that such methods were doing the Association harm, and the nurses might with advantage study the ways of the people a little more than they appeared to do." In reply to these comments, Mrs. C. P. Huntington, the president of the Association, observed that the nurses were all hospital trained, and they were not allowed to go beyond the doctor's orders. She believed that if the people more fully understood the methods of a nurse, they would better appreciate the work, and we have no doubt that she is right. But we are afraid that it cannot always be denied that district nurses are fussy. The hospital-trained nurse who takes up district work straight away, is naturally inclined to regulate her requirements by the resources to which she has been accustomed, and to be impatient if she finds broken jugs, cracked bowls, and lidless saucepans; whereas, if she has been through a period of district training she has probably learnt the valuable lesson that things which are desirable are not always essential. In other words, the best remedy for a tendency to fussiness in district work is to supplement hospital with district training.

### THE TRAINING AT CAMBERWELL INFIRMARY.

The training school in connection with the Camberwell Workhouse Infirmary has just completed its first three years, and the final test examination has been held by Dr. Bryant, of Guy's Hospital. Eight of the staff nurses, who were in the service of the Camberwell guardians at the time of starting the school, were allowed to enter for training and attend the full courses of lectures, and of these seven passed. Of nine probationers, seven passed, two only failing, and by so small a margin of marks that they will be permitted to stay on a fourth year, and enter for the final examination next year. The examiner, using the standard he adopts at Guy's, classed them as follows :—Three very good, five good, four satisfactory, two fair; these being the four terms used in defining results on their certificates. The examiner presented a good report to the guardians of the excellence of the teaching the nurses had received, and the guardians have accorded votes of thanks to the medical superintendent and the matron, congratulating them on the results obtained. Pending the opening of the new infirmary extension, the guardians have decided to pay all nurses in their service who have received their certificate £28 a year.

### FOREIGN NURSING INSTITUTIONS.

A "note" in our issue of August 16th has evidently been misunderstood by some of our readers, who complain that we did not more clearly indicate "the organisation in France" which supplied us with a text for our remarks. But our "sermon" was not intended as a criticism of that particular institution, it was meant to warn English nurses of the folly of joining foreign nursing institutions anywhere without the fullest inquiry. That there are others in addition to the organisation to which we alluded to be avoided, is shown by the fact that one of our correspondents who, fancying she has identified that originally cited, speaks of the two nurses as not by any means exhausting those who were dissatisfied, and names an entirely different institution in another part of France. As to the request that we should "more specifically single out places," we can only say that while we are always anxions to give information likely to be of use to our readers, and to call attention to any dangers which may beset the path of inexperienced nurses, there are limits to criticism which a journal must not overstep.

### A CENTRAL NURSING ASSOCIATION FOR SUSSEX.

It is formally announced that a central nursing association for the County of Sussex is to be established. This is the outcome of a conference which took place last November in London. The new organisation is to follow the lines of that already existing in the neighbouring county of Hampshire. It will, so far as funds admit, train women as nurses for local districts, and will facilitate correspondence, the superintendence and exchange of nurses, and "other matters," by providing one county centre in communication with London. There will be a county superintendent provided by Queen Victoria's Jubilee Institute, who will receive a salary of £110 a year, the Institute contributing £50 a year. The scheme has the advantage of influential local support, Lady De La Warr being chairman of the executive committee, and the Ladies Brassey, Leconfield, Mary Howard, Caroline Gordon-Lennox, Margaret Cecil, Henry Nevill, with Mrs. Wilberforce, wife of the Bishop of the diocese, her colleagues. We assume, moreover, that this list is not exhaustive, and that other active workers in the county will be invited to join the executive.

### A YORKSHIRE BENEFIT NURSING ASSOCIATION.

It is interesting to learn from Lady Margaret Bickersteth, the honorary secretary, that the Wilton Beacon Benefit Nursing Association continues to make good progress. Last year there were 789 members, and this year there are upwards of 1,000. The benefit subscriptions have also increased from £153 to £182, and the earnings from £145 for 127 cases to £197 for 147 cases. The expenses of the current year have been unusually heavy, owing to £109 having been paid for the cost of training nurses, but the Association has almost been able to pay its way, the balance on the wrong side being under £9. To meet contingencies, a sale of work on behalf of the organisation was lately held at Beechwood, Driffield, and about £300 was raised as the nucleus of a reserve fund. The position is therefore fairly satisfactory, though it may be hoped that there will be no need to draw on the reserve fund for ordinary outlay.

### SOUTHAMPTON NURSES' HOME.

The anticipations of the committee of the Royal South Hants and Southampton Hospital have practically been realised, and concurrently with the announcement that a tender for the erection of a nurses' home at a cost of £9,000 has been accepted, it has been intimated that the funds required have been obtained. This includes the £2,000 promised by Sir Donald Currie in the event of the total amount needed being collected by the Coronation.

### FREE TRAMS FOR DISTRICT NURSES.

It is urged by some of the friends of the nurses attached to the Leeds District Nursing Institute that they should be provided with free passes on the trams. This is a privilege which has been conceded in some other cases, but the Corporation of Leeds may consider that if it were afforded to the twenty-two nurses belonging to the Institute, other claims, at least equally good, might be made on their good nature. That nurses should have to use the tramways for travelling purposes is inevitable when they have to get over a good deal of ground in a short time, but as a matter of principle their fares ought either to be defrayed directly by the Institute for which they are working, or the amount of their salary should be regulated by the expenses which the committee know they will have to meet.

### TRAINING OF MIDWIVES.

The Association for the Compulsory Registration of Midwives request us to announce that a provisional committee has been appointed to obtain information on the present "training and supply of midwives" and we are asked to request all persons or institutions connected with this subject to send all information they may possessto Mrs. Schacht, 153 Cromwell Road, London, S.W.

### SHORT ITEMS.

Nurse Margaret Isobel Hellam, of the Portland Institute, Penrith, was married to Mr. Ralph T. Vincent at Christ Church, last week. She was attired in her travelling dress, and was given away by the principal, Mrs. McCane, being attended by four nurses in the uniform of the institute—blue dresses and bonnets—who carried bouquets of blue cornflowers and sweet peas. After breakfast at the Institute the bride and bridegroom left for Ireland.

### Lectures to Nurses on Medical Nursing.

By Eveline A. Cargill, M.D.Brux., L.R.C.P.&S.Ed.

LECTURE V.—DISEASES OF THE HEART AND CIRCULATION (*continued from p. 264*).

When a person "feels faint" make him sit down and bend his head forwards between his knees as low as he can get it; this allows blood to flow into the brain and may prevent his becoming unconscious. But if unconsciousness takes place he must lie down flat or with the head lower than the body. Do not allow people to crowd round, because a fainting person requires air. When he begins to come to, apply smelling salts to the nose, and give a little water to sip. If the faint continues loosen the clothes at neck and waist, chafe the hands, raise the arms, and, if necessary, expose the front of the chest and flap it with a wet towel. It is better not to give stimulants for ordinary faintness, but if any are necessary sal volatile is better than alcohol. In any case never try to force liquids down the throat till the patient is sufficiently conscious to swallow.

Fainting from heart disease is a more serious matter, and here stimulants are necessary, combined with the treatment of ordinary faintness. Artificial respiration should be employed if the faint continues long.

When to give stimulants is a difficult matter for a nurse to decide. She should always ask for definite instructions on this point, both for routine nursing and in case of emergency; what form of stimulant, whether brandy, whisky, or wine, and how much to be given in twenty-four hours. But sometimes she must decide quickly for herself, and for these cases she should have a knowledge of the action of alcohol, and whether it is likely to do good or harm in any particular case. Briefly, alcohol acts on the circulation, and on the nervous system, at first, and in small quantities, stimulating them (hence its use in heart disease), later, and with larger doses, depressing both the circulatory and nervous system. Because it stimulates the circulation, alcohol should never be given in hæmorrhage, where treatment must be directed to slowing and weakening the circulation. When bleeding is external it may be controlled by compression or by ligature of the cut vessel, but in internal hæmorrhage the circulation must be reduced as much as possible to promote clotting of the blood in the injured vessel. In nervous depression alcohol is better withheld entirely as the danger of becoming dependent on it is extremely great.

By feeling the pulse or stroke of an artery we gain information as to the manner in which the heart is carrying on the circulation, and the artery at the wrist is the most convenient for the purpose. A nurse should accustom herself to take careful and accurate note of the pulse both for her own guidance and to report to the doctor, and experience teaches what the different variations mean. The chief points to note in examining the pulse are: (1) The rate or number of beats per minute. Normally the heart beats about 72 times per minute, but may vary from 50 to 80. It is quicker in childhood and slower in old age. (2) Its regularity. In health the intervals between the beats are equal in length; in disease the intervals may vary in length, when the pulse is irregular, or a beat may drop now and again, when the pulse is intermittent. (3) Its volume. It may be large and full, or small, according to whether much or little blood is sent on at each beat; and (4) Its consistency. These last two points, viz. volume and consistency, can only be taught by experience. You must know what an ordinary pulse feels like before you can tell if one is too hard or too soft. There is a disease of the blood-vessels which makes them very hard and rigid, feeling like a cord. When arteries in the brain are so affected they are liable to burst and produce apoplexy from hæmorrhage into the brain. In mitral disease of the heart the pulse is quick, soft, and irregular; in fever it is quick, full, and bounding. Later, when the heart begins to fail, the pulse becomes weak, soft, and irregular. A wiry pulse is associated with abdominal inflammation, while in syncope it is soft and fluttering.

The great essential of treatment in all forms of heart disease when the circulation is failing is rest; rest of body and rest of mind. Patients with heart disease are apt to be nervous, anxious, and depressed, therefore the first duty of a nurse called to such a case must be to calm and soothe, to keep away all disturbing influences, all emotions, all noise. The room selected for the patient should be as far removed as possible from the sounds of the house; a sudden noise or a start makes the heart beat quickly even in health, much more does it affect a heart disabled by disease, even to the point of a fatal termination. So the patient must be secured quiet and freedom from anxiety or excitement of any kind; the visits of friends must be carefully controlled, and all disturbing topics of conversation avoided. The nurse should wear a confident and cheerful expression and request the friends to do the same, and endeavour to encourage and reassure the patient in every possible way.

The recumbent position diminishes the work of the heart, therefore lying in bed is advisable. There are conditions, however, in which breathing while lying down becomes so laborious that the effort of breathing in this position throws increased strain upon the heart. In these conditions, and in cases of heart disease when the lungs are much blocked the patient often finds it difficult to breathe lying down and must then be propped up in the sitting posture with a bed-rest and pillows, but he must not exert himself at all, and sudden movements of all kinds must be forbidden.

Plenty of fresh air should be admitted to supply the oxygen required, but draughts and chills must be carefully avoided. Food should be given in small quantities and frequently, and in a readily digestible form, as any overloading of the stomach would greatly embarras the heart. Treatment is also directed to drawing off water by the organs which eliminate water, viz, skin, bowels, and kidneys, and the nurse must be careful that the process is carried on without extra risk; for instance, the skin is encouraged to act by certain drugs and also by keeping the patient warm and sometimes bathing with warm water; but anything like exposure of a large surface of the body at one time must be avoided. The bowels are kept loose to drain off water and so relieve the stagnation in the veins, and also to avoid straining at stool, which is a great risk in heart cases; fatal accidents have not infrequently occurred from this cause. The nurse should also keep careful watch on the urine, and any marked variation in colour or quantity should be reported at once. Certain drugs which are given in heart cases are very powerful, notably digitalis, and the effect requires to be carefully watched when given in large quantities, as it has to be in severe cases. Digitalis strengthens the heart, and slows and steadies the pulse, but an overdose may produce an excessively slow pulse to 40 or less, with sickness and headache. It also acts upon the kidneys, increasing the amount of urine, but if the quantity of urine suddenly diminishes, this is also a danger signal, and must be reported at once.

### The New Annexe of the Erie County Hospital, New York.

BY AN ANGLO-AMERICAN NURSE.

Previous to the International Congress of Nurses I endeavoured to give intending nurse-visitors to the Buffalo Exposition a cursory glance over the Erie County Hospital and an epitome of its regulations. Since then some of my readers have had opportunity of seeing that our Transatlantic brethren have reached a fair stage of progression in other subjects as well as the shipping combine.

### The Western Consumptive Hospital.

They probably heard the history of the Western New York Consumptive Hospital, which was opened in 1896 and destroyed by fire early in 1900. It was attached to the Erie County Hospital, and, being a frame building, considerable difficulty was experienced in getting it insured; however, on payment of heavy premiums, that trouble was overcome.

Consumptives found there a delightful home, with spacious and airy wards; verandahs on all sides where it was possible for the sun to shine, floors highly polished, walls painted a pretty green, and ceilings in pink—pink, by the way, is the hospital colour. The sitting-rooms were fitted with rush lounges, self-adjustable reclining chairs, large polished tables—the drawers of which were well supplied with cards, draughts, dominoes, and other games with which to pass the weary hours. There were two small rooms for those in the last stages of the disease, a well-equipped pathological room, a special room for inhalations, and one for the exclusive use of the medical and nursing staff. But an accident occurred, due to two patients enjoying a quiet chat and prohibited smoke in the attic, which resulted in fire breaking out at midnight. Owing to the heroism of the doctors and nurses, every patient was removed unhurt and the fire department enabled to concentrate their whole attention on the flames. Happily, they prevented the fire from spreading, which was fortunate, for the building was connected with the nurses' home and the hospital proper by means of long covered corridors. The nurses' home, being parallel to the burning building, was therefore in imminent danger. However, the ill wind blew good, and with the aid of the heavy insurance and other appropriated sums of money, in addition to the advances which scientific research has made in the treatment of consumption, and the intense interest taken in this fell disease by the Houses of Parliament and Representatives, the Erie county now possesses the best-equipped hospital in the world for the treatment of tuberculosis in all its stages. The work of construction was commenced in 1901. Owing to the unusual requirements of such a building the most eminent experts were consulted, an advanced technical knowledge being necessary to plan a building conducive to the rapid convalescence of patients.

Having gathered all possible information the plans were drawn up by the architect. The constructing expenditure amounted to $49,900, and up to the present the cost of apparatus has been $4,000. It is an annexe of the Erie county hospital, and acknowledged to be superior to all similar institutions.

### The Accommodation for Patients.

The new hospital being completed and ready for occupation was opened for patients on March 31st. The two previous days were dedicated to inspection by visitors. The annexe is located south-east of the hospital proper, from which it is isolated as much as possible, yet connected by a lengthy covered corridor. It is two storeys high and built of hammer-dressed stone. To the south is a large meadow or recreation ground for male patients, and a similar ground to the north for female patients; both will be seeded and made beautiful by flower-beds prettily arranged; comfort added by means of garden seats and shelters with hammocks here and there. Accommodation is provided for 34 male and 16 female patients, the relative number of each sex being determined by medical statistics.

The lower floor is for men and the upper floor for women. The sanitary and drainage system is very complete and unconnected with the main hospital. To further prevent contagion the food for the consumptive hospital is prepared and cooked in the general hospital kitchen some distance from the new building, to which it is conveyed by a tramcar to the cellar, thence by means of an elevator or dumb waiter to the wards.

The main entrance leads into one of the two main corridors; to the left is a well-fitted dining-room for males, which accommodates 35 persons. Across the hall is the linen-room; here we mentally calculate whether the supply is adequate to the likely demand; next, we enter the inhalation-room, and it will be quite the right thing to express undisguised surprise and profound interest, for installed here are some of the most wonderful apparatus known in the treatment of this disease, including an actinolite instrument. The instrument generates powerful electric rays of from 20,000 to 30,000 candle-power, focuses them through a glass receptacle filled with water to cool them, centralises these in a powerful lens, from which they are thrown into the lungs to absorb the nodular growths (which are part of the disease) in these organs.

Another instrument in this room is an atomiser, which automatically sprays medicine over the organs affected. The wardrooms, each containing eight beds, are placed, as it were, in the four corners of each floor. The beds are after the most approved pattern, and each bed has its accompanying stand like those used in marine hospitals. On this floor are also two quiet-rooms for patients in the extreme stages of the disease. At the east end of the corridor is a solarium for morning use, and at the west end one for afternoon use. The patients are compelled to use the solarium at all hours of the day. In it every ray of sun reaches them, and by its use they can at all times enjoy Nature's elements. On the south side of the building is the day-room, which is fitted with everything likely to add to the comfort, amusement, and entertainment of the patients. The fact that a mind wholly contented and employed is an important factor in the progressive stages toward convalescence and recovery in tubercular diseases has not been overlooked in the equipment of this room, nor, indeed, throughout the hospital. There is also a broad piazza with garden-seats, where the patients can bask in the sunshine with books, papers, or games, or lose themselves in admiration of the many flowers, beautiful creations, perfected by art and attractively arranged, so that by contemplating their very brightness, sweetness, and variety one may forget at times the worries and troubles of life.

### The Nurses' Room.

At the junction of the two main corridors is the nurses'-room, or what is more frequently designated the medicine-room, for here the medicines are kept and dispensed, here the nurse reports to the doctor items relative to any patient that her judgment decreed should not be spoken in presence of such patient here the doctor issues, writes, and, if necessary, explains his instructions; and I sadly fear, from the numerous reported victims of Cupid, that here also the doctors and nurses find odd moments to discuss matters

### THE NEW ANNEXE OF THE ERIE COUNTY HOSPITAL, NEW YORK.—*Continued.*

quite unprofessional. However, being unable to speak from experience, I am not a very good judge; but I am quite sure that the doctors spend many half-hours, when they might enjoy a game of tennis, in helping each and every nurse with her studies, and explaining knotty points till she is fully conversant with the subject. Nor does this interfere with her duties, as all theoretical studies are done in off-duty hours. The upper floor for the use of female patients is a counterpart of the first, except that as less ward-room is needed this floor is fitted with well equipped bacteriological and pathological rooms, supplied with very powerful instruments of the latest patterns.

### Heating and Ventilation.

The system of heating and ventilating is also in accordance with the latest ideas of science, known as the direct indirect system, heating and ventilating being wholly independent of each other. Heating is accomplished by running hot air through a system of pipes, and ventilating by means of a fan in the attic operated by a motor. While the most approved of the latest scientific ideas have been installed, those which have stood the test of years and are still acknowledged to be valuable agents in treatment, are also provided and employed. Tile-flooring is used where permissible; corners are all rounded, and everything possible done to prevent any accumulation of dust with its attending propagation of microbes. Bath and toilet rooms are of course provided for the patients; also shower-baths, with floors of tile and porcelain walls, for patients who can stand them, and the usual baths for others.

The cellar is not a place where all kinds of lumber is secreted nor a grand central hot-bed for the cultivation of micro-organisms. Nothing whatever is stored there; the cellar is transformed into a kind of chief depôt for the regulating pipes and flues.

### Nursing in a Bible Mission Van.

BY A CORRESPONDENT.

At a very short notice I recently went to attend a patient who had an attack of diphtheria. The case was an uncommon one so far as surroundings and convenience were concerned, or, perhaps, one had better say "inconvenience." I had to take a long railway journey at the end of which there was a drive of six miles. I was met at the railway station by the only available vehicle for hire which the village boasted, something between a governess car and a dog-cart. The driver, a burly man, and his daughter, a girl of sixteen, were the only other occupants of the car.

### A Queer Place.

It was a clear bright night, with a tinge of frost, and the drive did not seem so very long and tedious as I had expected. "It's a queer place you be goin' to, Miss," was the driver's remark, "it be in a wood." I replied that I understood that was so, but that I knew something of the people I was going to. The driver also informed me that he "druv" the vicar to the station whenever such services were required. I arrived at my destination about nine o'clock, when I found a comfortable tea awaiting me, which I thoroughly enjoyed. The "destination" was a Bible Mission Van, and the "patient" was a young man about 30 years of age, who was in charge of the van in which he was lying ill. The attack had come on whilst travelling along the roads of a quiet county, and 30 miles from the nearest hospital. So a halt was made near a small village, and the van was drawn into a thickly-wooded forest, where the leaves at that time of the year formed a thick carpet. There was another young man assistant in the van, and also a boy from the village who helped with the cleaning, ran errands, carried water from the houses, in fact a good lad who made himself generally useful. The patient was very ill, and seemed to be slightly delirious; the assistant was prepared to leave for the night immediately on my arrival, having been invited to sleep at the vicarage, which was about two miles away. The boy also proposed to go to the village. On being asked if I "minded being left," I said I rather "did mind," and I thought it would be better if the boy stayed, although I was assured he would go fast asleep. It is certainly a nurse's duty to guard as far as possible against the spread of disease from the sick to the healthy, but I am afraid that the question of infection and risk to other people occupied a very vague place in my mind at this time. Still, the disinfectants I had brought with me were freely used all the time I stayed. Suffice it to say that the patient made a good recovery, and no one else took the disease. The doctor was our only visitor; he was most kind, and we looked forward to his never failing daily visit with his cheery words and genial manner as quite an event in that secluded spot.

### The Patient's Quarters.

The van, which was a very roomy and handsome one, had cost a good round sum to fit up, entirely at the expense of one gentleman. There was a door at each end, so that free ventilation was quite an easy matter, and I feel sure, had much to do with the success of the case. There were four windows fitted with pretty blinds, and these windows afforded good light, which is also curative. At night the van was lighted with safety oil lamps suspended from the roof. One end of the van formed a kitchen and could be shut off from the other part by a sliding door. In the kitchen there was a stove and oven, also shelves, cupboards, wash-bowl, and mirror. Every available space in the van was made into a cupboard with sliding doors which do not take up room. In the cupboards was kept a good supply of crockery, the pretty dinner service being the gift of a lady. One extra large cupboard was used for hats, coats, rugs, etc. Over the cupboards were book-shelves filled with books. The seats were very much like the seats in a railway carriage, with the exception that at night they were turned down to form a bed, of which the well-made cushions formed the principal part. The whole of the van was always most scrupulously clean, everything in its place, a picture of neatness, and this fact might commend itself to many people who are more happily circumstanced.

### Helping the Gipsies.

About a week before I left a number of gipsies arrived and encamped a little farther down the wood. We could see them quite plainly from the windows of the van. They had evidently come with the intention of remaining the winter in the forest, probably to replenish their stock of baskets and clothes pegs. While the patient was nearing convalescence I made them a pan of toffee and a number of apple tarts, and if one may judge by the way they disappeared they gave satisfaction. I received much kindness from everyone during my novel experience, not forgetting the old lady at the cottage where I hired a room, and who did everything she could to make me comfortable, and expressed regret at my departure from the Bible Mission Van to return to ordinary duties in a large city.

### A Visit to an Indian Leper Home.

BY A NURSING SISTER.

The medical officer in charge of the Home at Matunga for the most afflicted of God's creatures having courteously offered to take a friend and myself over the institution himself, we left Bombay soon after 6 A.M. one morning, about half an hour's run by rail bringing us to our destination. Matunga is one of the most remote of the outlying suburbs of Bombay. There are no carriages for hire, but the asylum bullock jutka is sent daily to the station to meet the doctor, and into this quaint vehicle we speedily proceeded to pack ourselves, a matter of some difficulty, requiring scientific manangement. The jutka resembles nothing so much as an oblong box on two wheels. The seats run sideways, facing each other, and are very near the floor of the carriage, and when we had arranged ourselves there was but little space between the roof and our sun-topees.

### The Grounds and Chapel.

The little bullocks trotted along at a brisk pace, the brass bells round their necks jingling merrily. In about twenty minutes we reached the asylum, which, surrounded by its own large grounds, is situated in a really beautiful spot. It was hard, as our eyes travelled over miles of soft green country stretching on all sides, to realise that we were still close to the noisiest, busiest, and most important city in the Empire of India. The grounds are extensive, the grass farm alone comprising 500 acres of land. They are ornamented in the immediate vicinity of the Home by beautiful old trees, under which ferns are picturesquely grouped, and brilliant flower beds. The trees provide an ideal refuge for numbers of singing birds, and their sweet notes had a special charm for ears accustomed only to the impudent chirp and the harsh caw of Bombay sparrows and crows. The first thing that attracted our attention was the sound of prayer, for a service was being held in the Roman Catholic Chapel. The chapel consists merely of a roof supported on pillars and open to the air and sunshine, only the altar and chancel being enclosed and railed off. In the open space several poor sufferers knelt devoutly. They were chanting a Litany with the priest, and ever and anon their plaintive voices filled the air with the solemn refrain. The sight was most pathetic, but one felt thankful that, cut off from all else that makes life worth living, these unfortunate people have still supplied to them the consolations of religion. Funds are being collected with which to build a chapel for members of the Anglican Church and Christians belonging to various Protestant sects. Not far from the chapel stands a Hindu temple, built on much the same plan. The railed enclosure which is kept carefully locked contains images of the most popular of the Hindu deities. A paid priest comes from time to time to administer the rites of this temple. His coming is the survival of the old East India Company's days, it having been one of its charities to support a priest in every temple. The religious needs of the Mohammedan patients are provided for with equal care, but we had not time to visit their mosque.

### The Female Patients.

The home contains beds for 370 lepers, 23 being accommodated in each ward. The buildings are all detached and at some distance from each other. Some of the wards have been specially built, others were originally barracks, which have been adapted to their present purpose. As far as possible the different castes and creeds are accommodated in separate buildings, one being especially set apart for the use of high-caste patients. The first we visited was for women and children. They all presented a tidy, and most of them even a cheerful, appearance. The ward was as neat and clean as possible, but the smell that assailed our olfactory nerves baffles description; penetrating and insidious, it invested each ward; wherever the lepers were there was this sickening odour. By far the greater number of the patients were suffering from tubercular leprosy, but we also saw many cases of the anæsthetic form. The tubercular is distinguished by an extraordinary thickening of the skin. If the face be attacked, this thickening imparts to the countenance a curiously distorted and lion-like expression. As a rule the extremities are first attacked. We thought that the fingers and toes of many of the patients had dropped off, but the doctor explained to us that this was not the case. The digits had contracted and shrivelled up, and the toe and finger nails were still visible on the shapeless stumps that had once been feet and hands. The anæsthetic cases are less painful to look at. In these the nerves are attacked, and a total loss of sensation results in the affected parts, which are marked by white patches. One nice-looking young woman was so afflicted in spine and shoulder. In others the patches appeared on face or hands. Such property as the inmates possessed was neatly disposed of in lockers beside, or in baskets under, their beds. Their brass *lotas* shone like gold, and so did the thalês (flat brass plates) from which the Hindus eat. Toys under many cots proclaimed that their occupants were children, but they were all at school save one, who, too young to learn, was playing about the ward. He was a chubby, healthy-looking boy, the child of leper parents, and had been born in the asylum. Alas! the inherited taint has shown itself already, and it is only a matter of time before the pretty baby face will become distorted and unrecognisable and the soft limbs lose their graceful contour.

### The Men's Wards.

Next we visited the men's wards; and here again we were struck with the air of cleanly cheerfulness that prevailed. The inmates are allowed to earn money in any way they can. One man is very clever in carving figures of Hindu gods, which he sells to the other patients. These figures, though crude in conception, rough in workmanship, and over-gaudy in colouring, are yet wonderfully well executed when one considers the crippled condition of the poor hands that did the work. In the Brahmin ward we saw a young man whose watery eyes and contracted lids showed where the disease had attacked him. Poor fellow! His eyes must remain open as long as he lives, for, sleeping or waking, he has no longer power to close them. But now the beating of tom-toms and clashing of cymbals, accompanied by men's voices singing a monotonous dirge, proclaims the fact that death has visited the lepers this morning. The body had been removed to the burning ground, but the funeral rites were not concluded. On inquiry we were told that the deceased had died, not of the leprosy from which he had been suffering, but from an attack of diarrhœa. Lepers are especially susceptible to changes in the temperature, and it often happens that a sudden illness brings their suffering to an end long before the natural course of the disease would do so.

### The School-Room.

In the school-room 32 boys and girls were happily at work. This building is bright and well ventilated; numerous doors open on all sides into the garden; coloured pictures adorn the walls, and in a large *almyra* (cupboard) with glass doors is displayed a tempting array of toys—notably a doll, much larger than a baby, which had been a Christmas

### A VISIT TO AN INDIAN LEPER HOME.—*Continued.*

gift from kind friends in Bombay. Many of the children were pitiful objects, especially a little lad of about 11 years of age, whose face and limbs were terribly swollen and diseased. The scholars looked wonderfully cheerful and were pleased to be noticed. A leper schoolmaster was in charge, he was instructing his pupils with considerable energy. We were rather startled to see a cane lying on the table beside him, but found that it was looked upon merely as a necessary piece of school-room furniture, for the master said naïvely, "It was only to frighten the children with, not for use." And indeed his almost fingerless hand could not have grasped it.

### Preparing the Meals.

Leaving the children to continue the tasks our entrance had interrupted we bent our steps to the kitchen. Here all was bustle and animation. Several cooks are employed, and they and the dhobeys are the only healthy people in the institution, all other forms of labour, save cooking and washing, being carried out by the lepers themselves. When we entered, preparations for the mid-day meal were in full swing. There are eight huge open fireplaces, and on each enormous cauldrons of *dhall** and vegetables were being cooked. The rice was already boiled and placed in large round baskets, ready to be carried to the different wards. It was very interesting to see the chaupatties being prepared. They are made of flour, water, and ghee (clarified butter), and in shape and size resemble a pancake. The Marathas call them *barleari*, and they form the chief food of the poor. One cook was kneading the huge lump of white dough; he passed small portions of it on to another, who flattened it out and partially baked it on large iron plates over the fire, and then in his turn passed it on to a third man, whose duty it was to lay the cakes on the red-hot cinders. When they had rested there a moment or two he gave them a dexterous turn, the other side was browned, and the chaupatti ready for eating. The cooks are Brahmins, but two Marathas are also employed to cook the meat which, of course, the Brahmins will not touch.

* *Dhall*, a small and very nutritious grain, which forms the chief food of the Hindus in Bombay.

### Lepers as Farmers.

The farm is a considerable source of revenue. The chief crops cultivated are Lucerne and Guinea grass, which finds a ready market. Three crops a year are reaped. Fruit and vegetables are also grown by the lepers for their own consumption. The water in which the soiled clothes are washed and all the sewage is collected into a large tank or filter, from this it passes into a second and a third, and in the third it is as bright and sparkling as if it had come from some pure mountain rill. The water thus purified is used to irrigate the farm and garden, and accordingly what might have been a source of danger to the healthy is turned by Nature's handmaid, science, to good account. A scheme is on foot to enclose two of these tanks in order that the gas generated may be utilised for lighting purposes. The men employed in the farm and garden earn two annas a day, the women one anna. This is a liberal wage when one considers that all their needs are fully supplied.

### Detention not Compulsory.

The segregation of lepers not being compulsory in India, the asylum is not guarded. No inmate of the home is ever discharged, but if he chooses to run away he is free to do so. Occasionally a man will grow restless and imagine that he may be cured elsewhere, but as a rule, having food and raiment, he is content. It is one of the characteristics of leprosy that the sufferer becomes a prey to languor and general inertia, and left to himself would do nothing to ameliorate his unfortunate condition. The Bombay Municipality gives a large grant towards the maintenance of this unique institution. The farm also brings in a considerable sum towards its support, and the voluntary contributions of the charitable public are solicited to make up what is lacking.

### The Nurse of the Future.

### A DREAM.

I was convalescent after a serious illness, and able to enjoy the society of the bright young nurse who had been chiefly instrumental in my recovery. She was essentially a modern nurse, and contrasted pleasantly with my memories of the prototype of the renowned "Sairey Gamp," who had ministered to me under similar circumstances 20 years ago. Then, convalescence was a dreary time, as I lay day after day listening to the eternal click of knitting needles, and the equally incessant clack of the lady's tongue, except, indeed, when her head fell forward on her breast, and gentle snores issued from her lips. Now, it was a pleasure to lie watching the white-capped head of my nurse bent over her book, from which her eyes were raised from time to time to see if I required anything. I was lying thus one afternoon, when looking up, she met my gaze.

"Well," she said, inquiringly.

"What is the book of such absorbing interest?" I asked.

"I am studying about aneurisms in Walsham. My friend, who has just gone in for her examination, had a question on them, and I am ashamed to say I remember very little of the subject."

"So much the better," said I, testily. "I don't see the necessity for nurses stuffing their heads with such things." (I am an old-fashioned man and have small belief in higher education.) "Let them learn how to make good beef-tea and leave aneurisms and like matters to the doctors."

"Come," said she, "you must not get excited. I will give you some nourishment, and then you shall try to sleep."

"That's always the way when you're getting worsted in an argument," I grumbled; but I meekly drank the beef-tea she brought and settled down to slumber.

"At any rate," I murmured as I fell asleep, "you know how to make it."

And as I slept, I dreamt. I was lying in bed with two doctors bending over me. A woman stood by in the cap and gown of a student. I heard the men call her "Nurse" and looked up in astonishment, for I had thought she also was a doctor. Presently the three moved away to the other end of the room, and I was left with night-shirt unfastened at the neck and untidy bed-clothes. Where was the nurse who should have put me straight? Consulting with the medical men over my case. I heard her voice joining in a discussion. By-and-by the doctors left, and then my small wants were attended to. But where was the tender womanly touch I dimly remembered in the past? Gone. My pillows were shaken up in a methodical manner, and I was laid back as if I were a log of wood. I dozed off for a time. When I woke my new nurse was bending over a table, on

THE NURSE OF THE FUTURE.—*Continued.*

which stood a microscope, together with a test-stand, and several learned-looking volumes.

"What are you doing?" I inquired feebly.

She did not look up as she answered. "Examining some of your blood to discover the approximate number of white and red corpuscles. The red seem very deficient."

"Perhaps some beef-tea might increase them," I suggested.

She rang the bell. Presently a maid appeared, and was ordered to bring up some beef-tea. It came. Luke-warm, watery stuff, with fat swimming on the top. I pushed it away.

"I'm afraid cook doesn't know how to make it," I said. "Would you be kind enough to show her?"

"I," she said in surprise. "I never made beef-tea in my life."

"Did they not teach you in hospital?"

"Oh no! all the cooking was done in the kitchen."

"What did you learn?" I asked, raising myself up to look at her.

"You mustn't sit up," she cried. "Don't you know you have an aneurism which is liable to burst at any moment?"

I lay down again.

"That is why I undertook your case. I am very interested in the subject of aneurisms just now. I wonder," she went on, reflectively, "what kind yours is. I think —— "

"Please don't," I entreated, "but tell me what you did learn."

"Oh, physiology, anatomy, and almost as much medicine as a medical student does. You evidently do not understand how advanced the nursing profession has become nowadays. We are no longer content merely to follow out a doctor's orders, but consult with them about the treatment of a case. We are taught as much theory as can be crammed into our heads during a three years' course of study. We must be conversant with Latin, and so able to understand all the prescriptions. Some of us think ourselves competent to write them. There is a stiff exam. to pass before taking our degree."

"Your degree!" I echoed, and sat up in astonishment, regardless of her previous warning. The effort was too much. My aneurism burst, and I awoke—awoke to find my own nurse bending over me, still holding "Walsham" in her hand.

"What is the matter?" she inquired.

I told her my dream.

She laughed merrily. "Your nurse doesn't sound very desirable. But you need have no fear. The doctors will never let us get as learned as that. Trust them not to teach us to usurp their prerogatives."

"I hope they won't," I replied, "for she was a terrible nightmare."

"I'm afraid your lunch has given you indigestion," said she. "It was rather early to start eating meat."

"No," said I, "you know it was nothing of the sort. My dream was caused solely by the fact of you studying 'Walsham.' Leave him alone, and read me the last new novel as an antidote."

And with all the energy I could muster, I flung the offending book across the room.

Nurse smiled, and gave me my medicine.

### A "Silent Hour" in Hospital Wards.

BY A NURSE.

"WHAT is the secret, sister, of the happy content of your nurses and patients?" asked a celebrated surgeon.

"I think, sir," she answered, "that it's due to our afternoon 'silent hour.' I never allow talking or amusement between 2 and 3 o'clock. This gives the patients a chance to sleep and the nurses to enjoy a hard earned hour of leisure."

Surely in the annals of hospitals there never was such a happy ward as this! I can answer for that, as I have done hospital nursing in most parts of the world and under all sorts and conditions of ward rules. How we nurses appreciated that daily silent hour for ourselves and our patients only tired workers can understand. It was a male surgical ward. Sometimes a new case grumbled a bit when "a poor man was robbed of his newspaper." But after a day or two he would fall under the influence of "nod" and volunteer to hide his book or paper, and our ward became as a garden of sleep a few minutes after the clock struck two. Early waking is the rule of all hospital wards, and sick people really need a restful interval in the long day. The bustle, routine, and busy hum that goes on so unceasingly is very trying to bad cases. Talkative neighbours in near beds, the passing to and fro of hurrying students, ward visitors, and nurses on duty bent, makes up an ever-moving panorama of activities. A silent hour for doze and siesta is a hygienic need in every sick ward, whether it be medical, surgical, male, female, convalescent, or children's.

It is the custom in all children's wards that I am familiar with to wind up the musical boxes, serve out the toys, trumpets, and noise-producers immediately the cots and wards have been "cleared up" after dinner. Some of those little patients have been awake and on the *qui vive* since daybreak. They are fractious and tired. A silent hour should be declared at two o'clock. Nearly all would sink into refreshing sleep. At three o'clock the small world would wake, alert and bright, to enjoy the good things from the hospital toy cupboard. I know it—for I have nursed much among children and have always enacted one quiet hour for my sick chicks, when noisy play and shouts and laughter were boycotted—just for 60 minutes.

The good sister from whom I learnt the "interval for silence" wrinkle in the days of my probation, was invariably angry with her staff if she found them "fussing about" in the forbidden hour. "You need a rest as badly as the patients. Enjoy your novel, or stitch at some fancy work. And don't stir from your nice cosy chair unless for urgent reasons or a bad case." She herself confessed to a daily forty winks in her own room off the ward And how delightfully fresh we all used to feel at three o'clock after our leisurely siesta in the drowsy, droning ward where the ticking of the clock was the only sign of life. The hospital porters soon learnt not to come knocking and shouting at our ward door during that magic, reposeful hour. None of the nurses can ever forget their sufferings when they were transferred to other wards where restlessness and noise prevailed more or less throughout the day. How our heads and backs used to ache from the constant bustle of our new surroundings. And how we longed day by day for that nice undisturbed "sit" for a whole hour in a comfortable old Windsor chair! It is not good for any sick person to be "in evidence" all day long. And there are usually some patients in a ward who are seriously ill and need periods of absolute quiet and stillness. One silent hour in the day is an easy rule to enforce. It soon comes to be regarded as a boon and a blessing by all the patients—to say nothing of the nurses.

### Everybody's Opinion.

[Correspondence on all subjects is invited, but we cannot in any way be responsible for the opinions expressed by our correspondents. No communication can be entertained if the name and address of the correspondent are not given as a guarantee of good faith, but not necessarily for publication. All correspondents should write on one side of the paper only.]

### PREJUDICE AGAINST NURSES.

"J. E. P." writes:—"I am afraid that some people are prejudiced gainst trained nurses by the following conversation I overheard whilst waiting for a chop to be served up at a West-End restaurant. Seated at the next table were two elderly men. I wore my nursing uniform and therefore I concluded that, as they were talking loudly, their remarks were made for my benefit. Their conversation opened thus:—

"How are they all at home?"

"Very bad; we have such a sick house; all the maids are laid up with sore throats."

"Oh, dear! I am sorry. Of course, you have a trained nurse?"

"No, indeed," was the reply. "Maria is not equal to waiting on a trained nurse."

### THE HOME SISTER'S AUTHORITY.

"A Liverpool Nurse" writes: The nurses in a certain institution in Liverpool do not wear out-door uniform, that being one of the conditions of the agreement. They supply their own clothes, and are, I presume, allowed to dress to please themselves. Under these circumstances can the home sister say what things must be worn on certain days? The case in point is this: on wet Sundays may they put on a sailor hat, or must they wear the only other one they have, with feathers or flowers as the case may be, which is quite unsuitable for the weather, and have it spoiled, because it is Sunday? Again, has the home sister any authority to stop nurses in the street, when they are with friends, and ask them when they cleaned their boots last? I should be glad if you would let me know how far we are under the control of the home sister in our off-duty time.

### PRIVATE NURSING IN SOUTH AFRICA.

"Mary Roberson" writes: I frequently see in the *Nursing Section of* The Hospital information asked for by nurses wishing to go out to South Africa. I was a nurse there for 11 years, and from my personal experience I should not think it good for quite young nurses, just having finished their training, to go, unless they had quite reliable friends to stay with, in which case the best plan would be for the friends to call upon some of the principal doctors and request them to place the nurse's name upon their list. A more experienced nurse, not much under 30 years of age, could write herself from England. I would willingly give the names of several I worked under, also any further information personally or by letter, or through The Hospital. My reason for the offer is a desire to be of service to any of my sister nurses, and to help my friends, "former patients," in South Africa to get good and reliable nurses. Of these there seems to be a scarcity, judging from the account I received a few weeks ago from a correspondent. She told me she could not get a nurse for less than four guineas per week. I seldom received more than three, but I had many valuable presents, and made what I believe to be lifelong friends. The nursing most required for private work is midwifery, monthly and sick nursing. Most surgical cases go into hospitals, as there are few conveniences in private houses. On the last point I would say that the conditions of life are by no means as smooth as they are at home. Black servants, either Kaffirs or Indians, are very unreliable. This means that much has to be done by a nurse which is outside her duty, but which she will try to do if she wishes to be a comfort in a house and her patients and their friends to be sorry when her time comes for her to leave them. I have no knowledge of hospital work in South Africa beyond the little one I organised and managed for a year, namely, Bired Hospital, Durban.

### THE LIGHT SIDE OF DISTRICT NURSING.

"F. P." writes: I was much amused on my round last week by the conversation which I had with one of my patients, and perhaps it will amuse other nurses also. My patient was a maternity case, a first baby three days old, and while I did my work we had a chat, relative, of course, to "Baby." "Where do you generally go to church?" I asked. "Oh, nurse, I am a 'West Lion'; me and my husband don't never go to church." "Then I suppose baby will be christened in your chapel?" "Dear me, no, nurse. We means to have her done properly in church." "Were you married in church?" "Oh, yes, we were married quite proper in church; but my husband he did teach in the 'West Lion' when he was single, but he don't hold with bein' married there. Now his eldest sister, she has been cook in a 'igh family for many years and she 'as seventeen servants under her; being as she lives with the gentry, she is a churchwoman and in course she goes to a very 'igh church as all the gentry does. There isn't one 'igh enough for her here, she is that 'igh." On another occasion I was very busy one day and the matron kindly went and did a case for me. On my visit the day after to the woman, she informed me she was sorry I was so busy but she was all right, "The cook had been and attended to her the day before." This rather surprised me as I could not understand her till she explained. "Law, nurse, I know'd it was your cook, for didn't her tell me as 'ow she made the 'soup.'" Matron afterwards told me she had introduced herself to the patient as the "superintendent!" Surely the poor are full of humour, and a district nurse need never be dull as she daily makes her visits amongst them.

### Appointments.

[No charge is made for announcements under this head, and we are always glad to receive, and publish, appointments. But it is essential that in all cases the school of training should be given.]

Bristol Royal Hospital for Sick Children and Women.—Miss Kate H. Todd has been appointed night superintendent. She was trained at the Bristol Royal Infirmary, and has since been head nurse at the Park Hospital, Lewisham, and at the North-Eastern Hospital, Tottenham. She was sister of the enteric wards at the Runchill Hospital, Glasgow, and later was sister of the children's ward at the Infirmary, Kingston-on-Thames. She has also had experience in private nursing on the private nursing staff of Fitzroy House Home Hospital.

Bristol Royal Infirmary.—Miss L. A. Baskett has been appointed home sister. She was trained at King's College Hospital, where she afterwards had charge of the ophthalmic wards. She has since worked as a private nurse, and latterly has acted as night sister at the Royal Hospital for Diseases of the Chest, City Road, E.C.

Cardiff Infirmary.—Miss E. Sproule has been appointed sister of the children's ward in this hospital. She was trained, and has since acted as staff nurse, in the same institution.

Culcheth Hall School, Bowden, Cheshire.—Miss Constance M. Bellingham has been appointed resident nurse. She was trained at the Salop Infirmary, Shrewsbury, and has been five and a half years on the staff of the Queen Victoria Surgical Home and Nursing Institution, Wolverhampton.

Hospital for Women, Liverpool.—Miss Evelyn Torbin has been appointed lady superintendent. She received her training at Charing Cross Hospital, and held the post of sister of a female medical ward in the same hospital for three years and a half.

Hull Sanatorium.—Miss E. Stevens has been appointed night superintendent. She was trained at the Hull Royal Infirmary, and has acted as charge nurse at the Brook Fever Hospital, Shooters Hill, for four years.

Kingston Hill Union Infirmary.—Mrs. Rose M. Liddell, Miss Lilian E. G. Shipway, and Miss Emily Howard have been appointed sisters. Mrs. Liddell was trained for three years at the Sussex County Hospital, Brighton. Miss Shipway was trained for three years at the Lambeth Infirmary, and remained for four months as staff nurse; for the past five years she has been attached to the Leicester Private Nursing Institution. Miss Howard was trained at the Poplar and Stepney Sick Asylum for three years, and remained for one year and four months as staff nurse. She has since been at the Royal Chest Hospital, City Road, London.

Longriggond Hospital, Lanarkshire. — Miss Stenhouse has been appointed matron. She was trained at the Belvedere Fever Hospital and the Victoria Infirmary, Glasgow, and holds the diploma of the Glasgow Maternity Hospital. Miss Stenhouse has subsequently been engaged in private nursing for the Co-operation for Trained Nurses in Glasgow.

Mill Lane Fever Hospital, Liscard —Miss Annie Edge has been appointed charge night nurse. She was trained at North Stafford Infirmary, Stoke-on-Trent, and has since been sister-in-charge at Middlesbrough Sanatorium, and has done private nursing at Bootle.

Princess Christian Cottage Hospital, Sierra Leone. —Miss L. Rhodes has been appointed sister at the above institution. She was trained at the North-west London Hospital and has since worked on the staff of the Mildmay Institution as private nurse, and as night sister at the Mildmay Mission Hospital, Bethnal Green.

St. Mary's Infirmary, Highgate Hill.—Miss Elizabeth Eyles has been appointed assistant matron. She was trained at St. Bartholomew's Hospital, London, where she was afterwards sister and assistant housekeeper.

Salop Infirmary, Shrewsbury.—Miss Flora Pegg has been appointed lady superintendent. She was trained at Guy's Hospital, and afterwards held the appointments of charge nurse at the New Hospital for Women, and at Netley House Nursing Home, and of sister at the Wolverhampton and Staffordshire General Hospital.

South Wimbledon Cottage Hospital.—Miss Mary Ridgway has been appointed staff nurse. She was trained at Birkenhead Infirmary for three years.

Union Infirmary, Stapleton.—Miss Martha Foster has been appointed sister. She was trained at the Fir Vale Infirmary, Sheffield, and subsequently became superintendent nurse at the Easington Union Infirmary, and sister at the Union Infirmary, Wakefield. She holds the L.O.S. diploma, and has been engaged in private nursing.

University College Hospital.—Miss Dora Finch has been appointed matron. She was trained at St. Helen's Cottage Hospital, Lancashire, and St. Bartholomew's Hospital, London. On the completion of her three years' training in the latter institution she became staff nurse, and was subsequently night superintendent and sister. Since February, 1899, she has been matron at the New Hospital for Women, Euston Road, London.

### Wants and Workers.

The matron of the St. Clement's Maternity Home, Fulham Palace Road, S.W., will be glad to receive a go-cart or perambulator for the use of a motherless baby.

Nurse Fricker, Parish Nurse, 6 Summer Road, North Peckham, London, S.E., asks if kind friends will send old story books to lend to girls between 14 and 16 years of age, and old linen for the use of poor patients in the parish.

### For Reading to the Sick.

### THE LIFE OF FAITH.

Let us do our work as well,
Both the unseen and the seen;
Make the house where God may dwell
Beautiful, entire, and clean.

Else our lives are incomplete,
Standing in these walls of Time;
Broken stairways, where the feet
Stumble as they seek to climb.

Build to-day, then, strong and sure,
With a firm and ample base;
And ascending and secure
Shall to-morrow find its place.

Thus alone can we attain
To those turrets, where the eye
Sees the world as one vast plain,
And one boundless reach of sky.

*Longfellow.*

Do not let us think that eternal life is *yet to begin* for us. It began at our baptism. It began, consciously, the very first moment we realised what it was to believe in God, to serve Him, and to pray to Him. It is going on now. Do not let us think that there is a hard-and-fast line drawn by death—what we call death—and that we are on one side of it and all the saints of God on the other.

We *are* in eternal life now. We can only forfeit it by our own fault. We have got our feet on the lowest grades of the heavenly staircase that reaches up to the very throne of God. How this thought should ennoble our daily life and work! How it should make us shrink from everything deadly and corrupt! . . . It is because our lives are Godward that they are eternal.—*Elizabeth Wordsworth.*

We only live, in any true sense, as we are filled with the heavenly magnetism. "Thou hast made known to me the ways of life; Thou shalt make me full of joy, with Thy countenance," says the disciple. Here is the true gospel to live by. There *are* "ways of life"; and through toil and trial they shall be reached. The one is eternal, the other temporal. It is unwise to lay too much stress on the infelicities of the moment. For one mounts to eternal life now, not in some vague to-morrow, but to-day. Eternal life is a condition, not a period. Live in immortal energies, in noble purpose, in true lift of soul, and one lives at once and here the immortal life. His soul has already put on immortality.—*L. Whiting.*

Therefore to whom turn I but Thee, the ineffable Name?
Builder and maker Thou of houses not made with hands!
What?—have fear of change from Thee who art ever the same?—
Doubt that Thy power can fill the heart that Thy power expands?
There shall never be one lost Good! What was, shall live as before;
The evil is null, is naught, is silence implying sound;
What was good, shall be good, with for evil so much good more;
On the earth the broken arcs,—in the heaven a perfect round!

*Browning.*

### Echoes from the Outside World.

### The King and His People.

It having been brought to the notice of the King that there was a general wish for the reproduction in facsimile of the letter which his Majesty wrote on the eve of the Coronation, expressing thanks to his subjects in all parts of the world for their sympathy during his severe illness, he commanded the Home Secretary to take the necessary steps. A facsimile of the document, which is entirely in the Sovereign's handwriting, is therefore now obtainable. The King has also sent a letter to Lord Kintore, as representing the Gordon Highlanders' Memorial Institute in Aberdeen, in which he expresses his desire to congratulate Captain Brooke on the energy which he has displayed in endeavouring to form the Institute, and the success which has attended his efforts.

### The Shah's Visit to England.

The Shah completed his visit to England on Monday, after a stay of a week. He has an intense dislike of the water and went round the Caspian Sea rather than cross it in a steamer. As, however, he could not reach England by land, he took the shortest sea-passage from Calais to Dover, and insisted that the boat should not be driven at too high a rate of speed. He was received at Victoria by the Prince of Wales, and a large crowd assembled to witness his arrival. The Shah is very much like his father, but the most striking feature about him is the sombre melancholy of his eyes. His expression, though cold, is gentle, and there seems to be no intense fear of him amongst his suite, such as is usually supposed to exist in the Courts of Eastern potentates. An escort of Life Guards accompanied the Royal guest to Marlborough House. In the evening there was a State banquet at Buckingham Palace in his honour. Next day, after a full-dress reception in the morning, the Shah drove in the afternoon to Madame Tussaud's, where he hoped to have seen the effigy of his late father, but it had been removed. He was much gratified at finding his own waxwork presentment. In the evening he went to the Empire Theatre. Although he seemed interested, he never smiled, even when the rest of the house was convulsed with laughter. On Wednesday the Shah repaired to Portsmouth to lunch on board the Royal yacht. He dressed, as usual, in the train, and his breast and his "ko-o-lah"—or fez—were resplendent with jewels when he arrived. He remained on the *Victoria and Albert* till four o'clock, and on parting from the King was seen to smile for the first time. On the return journey he stopped the train because it was going "too fast." On Friday he visited the factory of Sir Hiram Maxim, and was extremely keen to learn all that he could about the various guns. He had the mechanism of the Maxim gun explained to him, and then sat down and fired off several rounds himself, nodding repeatedly with satisfaction. In the afternoon he visited the scene of the Coronation at Westminster Abbey, and went later on to the Hippodrome, where he was in much better spirits, laughed at the comic portions of the entertainment, and appeared to find pleasure in conversing with Prince Arthur of Connaught. On Saturday he attended a review on Woolwich Common, which he seemed to appreciate, but was so bored afterwards at the Arsenal that the programme had to be cut short. In the evening he had a private dinner-party at Marlborough House, and on Saturday he went to Windsor to shoot in the park and to see the King's home. In the evening he went to the Crystal Palace to witness the fireworks.

### The French Religious Orders.

The expulsion of the French religious orders has been accomplished. In this connection it has been noted with satisfaction by many of our neighbours that the King and Queen of England last week visited the colony of French Benedictine Sisters who have recently established themselves at Northwood in the Isle of Wight. The Archduchess of Braganza is a member of the sisterhood. In view of the historical importance of the movement it may be interesting to recite the facts of the case. The Religious Associations Law, which was passed in 1901, requires religious establishments to be registered and authorised by the State. On October 3rd, last year, the last day for sending in applications for authorisation, out of 16,468 religious establishments in the country only 2,001 male and 6,799 female communities made the necessary application, of the others some left the country and sought refuge in Belgium, America, Switzerland, the Channel Islands, and elsewhere, while the rest remained in France and neglected to apply. On July 15th the Government ordered the closing of 2,500 establishments and they have since taken drastic steps to enforce the order. M. Combes has, in fact, only succeeded in getting the law executed by employing soldiers and gendarmes to evict female communities.

### The Largest Vessel in the World.

The *Cedric*, which was launched last week from Belfast and is intended for the passenger service of the White Star Line Company, is the largest vessel in the world. Her length is 700 feet, her beam 75 feet, and her depth 49 feet, and she weighs 21,000 tons gross. The *Cedric* has nine decks, and is built on the cellular double-bottom principle. She is divided into so many water-tight compartments, exceeding all official requirements, that a maximum of security is obtained. As to her accommodation, there are quarters for 3,000 passengers, and a crew of 350. In addition to the ordinary state rooms, there are suites consisting of bed, sitting and bath-rooms for families. There is practically a new feature in ocean liners, namely, single berth state rooms. The first-class accommodation is all arranged amidships on the upper decks, the number of such passengers thus provided for being 365. The grand dining saloon is on the upper deck, is 75 feet long, and will seat 300 persons. Aft, on the upper and bridge decks, there is provision for 160 second-class passengers, including saloon, smoking-room and library; and on the upper, middle and lower decks there are numerous separate cabins and open berths for third-class passengers. The *Cedric* is expected to make her first voyage from Liverpool to New York in November.

### Accidents of the Season.

A particularly sad disaster happened near Filey, in Yorkshire, at the end of last week. Three children of one family, and two little cousins belonging to another, ranging in age from twelve to three, were playing on the sands in the afternoon, when they were surrounded by the tide and drowned in the sight of the two mothers, who waded into the water breast high, in their efforts to save their children. The two eldest girls held up the little ones in their arms until they themselves were swept off their feet by the waves. Two lamentable accidents have also happened during the week on the Alps. Two gentlemen from Aberdeen, with a couple of guides, ascended the Wetterhorn, when they were overwhelmed by an avalanche, hurled a distance of 1,200 feet, and finally flung on to some rocks. One traveller and one guide were killed at once, the others remained, much injured, for several hours before they were conveyed back to hotels. Later on another fatal accident occurred on the same mountain. Two brothers named Fearon, and two guides, were all killed by lightning; the two whose bodies were recovered being completely blackened. Both guides leave wives and children.

### A Book and its Story.

### MR. BARING GOULD'S NEW NOVEL.*

In "Miss Quillet" Mr. Baring Gould tells the story of a girl of good family who, finding home-life intolerable, seeks distraction as a probationer in a London hospital. The immediate cause of dissatisfaction is her incapacity to cope with the domestic difficulties of a household consisting of three sisters younger than herself, her father, Colonel Quillet, of the Grenadier Guards, who failed entirely to exercise the control over his family and a staff of insubordinate servants, which he may have held over his regiment. Anne, the heroine, decides that further excitement and more variety are necessary to her existence, and takes up nursing as a means to this end. On the first page she appears indirectly in a dialogue between a country surgeon, and an aggressive sister of Richard Marmaduke the patient, for whom a nurse is required. The case is one requiring care and vigilant attention, rather than technical professional knowledge. Richard Marmaduke, partner in a firm of solicitors, had, beside his practice, an interest in science, as a recreation. Chemistry had a special fascination for him, and in experimenting one day in his laboratory, an explosion occurred which seriously affected his eyesight. The influenza epidemic was raging and nurses were at a premium. Mr. Tolcher held a telegram in his hand. "What is to be done?" he asked of Mrs. Basileg. "The reply to my wire says, 'Can send no trained nurse; probationer alone available, if no infectious case; wire if required.'" Mrs. Basileg would not hear of a probationer as a nurse to her brother. But two other institutions having been applied to with the same result, she finally left Mr. Tolcher to decide, not without protest and much inconsequent chatter on her part. "Really," said Mrs. Basileg, "I do wish that Richard had taken up with some other pursuit than chemistry, which always blows people up sooner or later." "Madam, it is vain to wish—we must act." "It's all nonsense," exclaimed the angry lady. "You can't go in a 'bus or on the Metropolitan Railway but you encounter nurses or art students; they swarm as thick as midges in autumn. Where are they all now? I mean the nurses, not the art students." "They are not at the hospitals or institutions to which I have applied," said Mr. Tolcher. "No, they are everywhere but where they are wanted, rushing about with their veils flying behind them like mosquitoes. However, no other alternative presenting itself, the services of the probationer have to be accepted, and Anne Quillet comes on the scene in person. Her first interview with Mr. Tolcher, after receiving from him instructions for the care of her patient, concludes thus: "You understand me?" "Yes," replied Anne. "How long am I to be shut up in the dark with him?" "How long?" asked the surgeon, in surprise. "How long? Why, of course, as long as is necessary." He stood looking at her questioningly for a moment, and then said abruptly, "I don't like that." "Do not like what?" "What you said on coming into the sick room." "What was that?" "You asked how long you would have to be in there." "It is not a particularly attractive chamber." "That it is not. A nurse if she is worth her salt, thinks of her patient and not of herself. Do not disappoint me." "Remember I am only a probationer." "You are on your probation then, now. I leave a most important trust in your hands. Mind, nurse, it is a trust." Left alone in the darkened room, having ascertained that her patient slept profoundly under the influence of the drug that had been administered by the doctor before leaving, she finds the situation intolerable. To her undisciplined nature the prolonged watch without a novel or even a newspaper seemed unendurable. "In herself she had no resources." Now, in the desperation of loneliness, she looked round the sparsely furnished bachelor's room for anything in the form of literature, which might give temporary diversion. After an inspection, difficult in the dim light, she found nothing which suggested alleviation. "Anne was unaccustomed to needlework, or she might have occupied herself in darning a hole in the eiderdown quilt thrown off by the patient, who with returning consciousness was becoming restless. When any of her own garments required to be taken in hand she sent them to a seamstress; till they were unfit to wear, she fastened them together with pins."

But Anne was not without resource, of a kind, for we read, "I must have something to read, even if I burn my eyes out over it." Then it occurred to her that the drawers might be lined with newspapers, and opening one she pulled out the sheet which it contained. But its perusal was interrupted by the voice of her patient inquiring who was in the room. "It is I, your nurse." Then ensues a conversation in which Anne hears something of his past life and future hopes, for the present frustrated. The following morning when Mr. Tolcher arrives he concludes from the feverish condition of the patient that something has happened to disturb him. "Did he talk," asked the doctor, "did you draw him on to speak much?" Anne hesitated a moment, flushed up, and replied, "Yes, we did have some conversation. For my part I do not see how it could be avoided. It is intolerably dull for him, as it is for me, to spend hours interminable in the dark."

"You are here not to pass your opinion but to receive orders," retorted the surgeon. "Please further to remember that you are here not for your amusement, but to fulfil a duty."

How Anne failed in the fulfilment of her duty, how grievously she erred in raising the blind and omitting to lower it again before she left the room and went out for her walk, of the distractions she encountered out of doors which made her oblivious to her patient, who through her carelessness lost the sight of one eye and imperilled the vision of the other, the reader will discover in Mr. Baring Gould's story. Her character and the dialogue in many places is, we hope for the sake of Miss Quillet's sisters on probation, and their patients, mere parody. But as an exceptional specimen of a type which perhaps the author had in mind, Anne Quillet may not, alas! be overdrawn. She belongs to the class of educated, well-born, but undisciplined women who for lack of interest in the duty that is nearest, desert the homes which they should adorn, to take up a profession from sheer *ennui* which requires the exercise of the very virtues in which they are deficient—patience, sympathy, gentleness, and self-control — virtues which a nurse's life may develop, but which it assuredly will not produce if the seed has not been sown, and exercised first, in home life. There is no vice in Anne Quillet, only culpable thoughtlessness, and she works out her redemption later when, aroused to a sense of what life should hold and of what her own lacked by the scathing criticism of a man friend who was speaking to her of the neglect of the nurse who caused the loss of eyesight to Richard Marmaduke. "To think that one of the sex should act thus! It fills one with disgust. Thank God, that there is not one in a thousand who would have so forgotten herself and have done what that creature did." "Do you know her name?" asked Anne, in a muffled voice. "No, I do not want to be told it." "You are quite right," said Anne slowly, her eyes fixed on the ground. "No word is too strong. She was a dishonour to womanhood."

* "Miss Quillet." By Baring Gould. 1 vol. Illustrated. Publishers: Methuen and Co. 6s.

### Notes and Queries.

**The Editor is always willing to answer in this column, without any fee, all reasonable questions, as soon as possible.**

**But the following rules must be carefully observed:—**

**1. Every communication must be accompanied by the name and address of the writer.**

**2. The question must always bear upon nursing, directly or indirectly.**

**If an answer is required by letter a fee of half-a-crown must be enclosed with the note containing the inquiry, and we cannot undertake to forward letters addressed to correspondents making inquiries. It is therefore requested that our readers will not enclose either a stamp or a stamped envelope.**

*Maternity Case.*

Will you kindly tell me where a thorough training in midwifery can be obtained and the lowest cost. My means are limited and I should be grateful for advice.—*M. E. H.*

The British Lying-in Hospital, Endell Street, St. Giles, W.C., inclusive fees, £22 10s.; the City of London Lying-in Hospital, City Road, E.C., fees the same. The East End Mothers' Home, 394 Commercial Road, E., fees (not inclusive) £15 15s. These are the cheapest fees in London and include board-residence. You might do better locally; ask your medical man or district nurse how you can be most cheaply prepared for the L O.S.

A nurse having two years' general training wishes to get maternity training free. Please tell her the best way to do this.—*L. P.*

The only way is to make a private arrangement to give services in return for training. There are several private homes which might do this, and your best plan is to advertise for such.

I am anxious to take my L.O.S. Is it possible to do so whilst district nursing, and, if so, what steps ought I to take? I hold a three years' certificate. What books should I read up meanwhile? —*E. A. M.*

You can prepare for the theoretical part of the examination whilst district nursing, but it would not be advisable for you to attend the practice whilst you are engaged with other nursing. Your best plan would be to write to the Secretary of the London Obstetrical Society for a syllabus, and then to ask your medical man or a qualified midwife to superintend your study. During a holiday you could do the practical part of the work, and then present yourself for examination at the first opportunity. Books will be recommended by your teacher.

Will you kindly tell me if six weeks is sufficient training for a midwife? Are there any hospitals that train pupils in this time?—*F. J.*

The usual training for a midwife is three months. See replies above.

Will you kindly tell me if there is any place in London where I could be trained for a few weeks in maternity nursing, receiving board and laundry for services?—*A. W.*

Certainly not, for the simple reason that your services would be worth nothing until after you were trained.

Can you kindly tell me if a nurse who has been trained on the district in maternity nursing, and has a certificate, could enter for the L.O.S. examination without further training?—*Nurse H.*

You would probably have to be prepared in the theoretical part of the subject. See replies above, and write to the Secretary of the L.O.S. for advice on your special case.

Will you kindly tell me where I could receive the loan of bones to study for my midwifery examination?—*Nurse E.*

The Secretary of the Midwives' Institute, 12 Buckingham Street, Strand, W.C., would be most likely to help you in this.

I am a maternity nurse, and a few weeks back a lady sent for me and engaged me to nurse her. My return railway fares cost me 2s. 2d., and there will be my fare there and back when I go to nurse her. I should be thankful to know if it is usual for a private nurse to pay her own fare.—*E. E. H.*

The payment of the fare when you go to attend is naturally a matter of arrangement between the nurse and the lady. You can get out of the difficulty in future by having your terms printed on your card and noting that you expect to have your fares paid. But in regard to the railway fare on the occasion of your preliminary visit, which we presume you made at her request and for her convenience, we think the lady ought to pay it.

Will you kindly tell me if there is any hospital where a maternity nurse can get a short training in general nursing with a salary?—*N. A. H.*

Advertise, as some of the smaller hospitals might possibly give you what you want.

*Abroad.*

(128) Do you think that I could obtain charge of a patient, going to Australia or New Zealand, who would give me a return ticket for my services? Would you kindly tell me the best time to advertise, and also mention if you know anything as to the kind of nursing most in demand in these colonies?—*Nurse and Nurse G. I.*

Advertise at any time in the medical papers for a patient. You can either ask for a return passage, or charge sufficiently to pay your own return journey and out-of-pocket expenses. Good nurses can always find employment when they become known, but there are plenty of nurses of all kinds in the colonies you name, and their fees are not higher in proportion to the cost of living than they are in England.

Will you kindly tell me where I can obtain information which will enable me to obtain an appointment as mental nurse in Paris, either in an institution or in a private family?—*E. G.*

Your best plan would be to write to the Matron at the Hertford British Hospital, Rue de Villiers-Levallois-Perret, Paris.

Can you give me any information respecting a hospital in Auckland, New Zealand? I am a trained nurse, and anxious to get into one.—*Zilpa.*

Apply to the Lady Superintendent, Auckland Hospital, New Zealand, for information.

Would you kindly give me the names and addresses of nursing institutions in Cairo, Algiers, or Tunis?—*L. A. W.*

Miss Cutler, Victoria Nursing Home, Cairo, might give you information as regards private nursing institutions there. We have no particulars of such establishments in Algiers, etc.

Will you kindly give me the name and address of the Nice Nursing Institute?—*K.*

The Nice Nursing Institute, Villa Pilatte, Avenue Desambrois, Nice.

Will you kindly tell me if it would be possible for a trained nurse to obtain work in Seville or elsewhere in Spain? Are there any English doctors to whom she could apply?—*Scotchwoman.*

Write to the English Vice-Consul at Seville, enclosing stamps for reply.

*Army Nursing Reserve.*

(129) Will you kindly tell me if nurses can still join the Army Nursing Reserve, and if it is likely that any fresh ones will be sent to South Africa?—*Matron.*

You had better write to the Director of Queen Alexandra's Imperial Military Nursing Service, 18 Victoria Street, S.W. Although it is not probable that nurses will be wanted for South Africa, yet if a nurse is anxious for national service abroad she can keep her name on the list—that is always supposing she is accepted—and she will then be in a position to volunteer in any future emergency.

*Hospital Training.*

(130) Will you kindly tell me what qualification I should require in order to become a district nurse? I hold the certificate of the Queen Charlotte's Hospital.—*F. I. U.*

You will require at least a year's training in general nursing and six months' in district work. Two years' general training would be better still.

I am 19 years of age and wish to begin training in a good hospital when I am 21. Would it benefit me to apply to have my name put on the books now, as I have been advised? I know that 21 is considered young. Would it affect my professional career if I took a post as companion-governess in the meantime?—*S. H. W. and F. S. and H. W. M.*

It is no use putting your name on the books before you are ready to begin work at 23. By no means.

I have had one year's training in a general hospital and six years' experience in private nursing. I am anxious to enter the Navy Staff of Nursing Sisters, but find that a three years' certificate is necessary. Could I get into any other hospital for two years' supplementary training, or take a minor post in a hospital for that time, and so earn my three-year certificate?—*S. M. K.*

It is extremely difficult to get into this service, because there are so few vacancies. You had better write to the Director-General, Medical Department of the Navy, Northumberland Avenue, W.C. State your case and ask if it is any use your training for the staff.

**Standard Books of Reference.**

**"The Nursing Profession: How and Where to Train." 2s. net; post free 2s. 4d.**

**"Burdett's Official Nursing Directory." 3s. net; post free, 3s. 4d.**

**"Burdett's Hospitals and Charities." 5s.**

**"Hospital Expenditure: The Commissariat." 2s. 6d.**

**"The Nurses' Dictionary of Medical Terms." Cloth, 2s.; leather, 2s. 6d. net.; post free, 2s. 8d.**

**"Burdett's Series of Nursing Text-Books." 1s. each.**

**"A Handbook for Nurses." (Illustrated). 5s.**

**"The Physiological Feeding of Infants." 1s.**

**"The Physiological Nursery Chart." 1s.; post free, 1s. 3d.**

**All these are published by the Scientific Press, Ltd., and may be obtained through any bookseller or direct from the publishers, 28 and 29 Southampton Street, London, W.C.**

### Travel Notes.

By Our Travelling Correspondent.

### CVII.—WHAT WE CAN SEE FOR FIVE POUNDS.

### Some Châteaux in the Meuse Country.

In my paper on Dinant I purposely said nothing of the ancient manor-house of Veve-Celles, which you often hear spoken of as Weve. It is a place so interesting, though totally bare and empty, that I felt I must not relegate it to a few final lines in an article on a different place.

Guide-books hardly mention it, and perhaps that is natural, for if you did not know something more or less correctly of its private history it would not appeal to you much more than many other dismantled domestic buildings.

### The Château of Veve-Celles.

The history (but imperfectly known) of this weirdly interesting house was thus told to me by an artist of some note staying in the same hotel, and the mysterious chord which thrilled through his account sent me the very next day to explore for myself.

It seems that about 100 years ago the owners suddenly left their beautiful home, never gave any explanation of their conduct, and never returned. Naturally the popular idea is (though probably quite erroneous) that some terrible crime was committed there which made the house intolerable to the occupants. The rooms seem to have been so hastily deserted that they are not altogether dismantled, several mirrors and some tapestry still clinging to the walls.

My friend lent me "Through the Ardennes," in which the gifted authoress's account of this strange place occurs, and I think I cannot do better than give you her own words, though I must, unfortunately, cut them down very much. After visiting the place I felt how absolutely she had entered into its spirit, and how exactly she described the strange feeling of weird intelligence which I myself felt to exist between me and the house with its silent yet eloquent walls.

"The present castle dates from the beginning of the fifteenth century. . . . This passage gives a half-turn into a five-sided courtyard; grass and nettles grow everywhere, and brambles cling over a rusty-iron railing beside a flight of broken steps; there are doorways on every side, but the side under which we enter the courtyard is much longer than the others. . . . Across it runs a quaint open gallery of two storeys, partly screened by a half-timbered parapet, from which the yellow plaster is mouldering away, showing patches of the red brick which it has covered. From each gallery a series of rough black arches support the upper storey. The roof rises steeply, crowned with elaborate chimney stacks dotted with tiny dormer windows. . . . The first large room was strewn with books—English, French and Italian, Spanish, Latin and Greek. . . . All this was left to perish in the dust and squalor of the place companions of loathsome things; for

'The centipede along the threshold crept,
The cobweb hung across in mazytangle,
And in its winding sheet the maggot slept
At every nook and angle.'

It was all very ghastly and desolate, and we felt glad that a proposal to dine in one of the deserted rooms had not been carried out. . . . However gloomy and lonely, I wished to *be* alone in the old place. Truly Hood's verses might have been written here.

'No other stir or sound of life was there,
Except my steps in solitary chamber,
From flight to flight from hurried stair to stair.'

Each flight of these dreary stairs carried me into a deeper sense of mystery, till I expected to find lurking in some inner room or forgotten corner, the witness of the past, the clue to the strange doom that has fastened on this fine old mansion. Near the top of the first staircase a curious tesselated pavement led me to the chapel. . . . The crucifix still stands on the altar, and there is less squalor here than elsewhere. I went on to the bedrooms; in one of these, entered through a smaller room, is a portrait, or the remains of one, over the empty hearth. It is painted on panel and the injuries it has sustained give a strange weirdness to the expression of the face; one might fancy 'The old ancestral spirit knew and felt, the house's desolation.' There was no bed in the alcove. . . . I wondered what was the story of this room and of the dressing-room within it. . . . I turned from the window and looked round the room; there was nothing to see, and yet it felt as if there might be found something by looking for it. Something—seemed to make thought leaden and to clog all power of enjoyment. I left this room and explored the long galleries, with suites of deserted chambers leading from them. I saw other pictures, other traces of habitation, a cracked mirror on the wall, a true lover's knot in stucco above a mantel-shelf; but none of these had for me the weird fascination of that inner room with its tapestried *cabinet de toilette* I felt that I would not dare go into it in the gloom of the evening. I was glad to leave it finally, for I found myself drawn back there again and again by some secret fascination, till I grew almost sure the walls could mutter some dark story, buried under the cobwebs of the past. . . . It had become very dark while I had been groping about upstairs, and soon a furious thunderstorm broke over us. The stillness that followed was awful, and then in the dead hush there came the faint toll of a bell—only one stroke—as if a phantom in one of the far-off chambers had been summoned home. It proved to be only a prank of our youngsters who had found the bell-tower. The storm was over, and we started home to reach Dinant before dark.

'O'er all there hangs the shadow of a fear
By sense of mystery the spirit daunted
Says plainly as a whisper in the ear,
The place is haunted!'"

### How to go to Veve-Celles.

I have given you Mrs. Macquoid's own words, though sadly abridged, feeling that none of mine could put before you so powerfully the strange obsession that emanates from Veve. If you walk there it is about six miles each way, but a carriage is not expensive.

### Walzin and Montaigle.

I have left myself but little room to speak of these places. Walzin, as I told you before, is not far from Dinant, and is chiefly interesting from its magnificent situation and that it originally belonged to the de la Marck family. Montaigle is full of interest. It once belonged to the Berlaymonts and was the scene of a grim family tragedy in the time of the Crusades. I cannot remember where I read the story, but probably in "Through the Ardennes," which is a useful book to study before you leave England, but having been written many years ago it is out of date as to means of locomotion. Many new lines of rail have been constructed since those days, to the impoverishment of the picturesque, but to the gain of our pockets.

